# Three‐dimensional in vitro maturation of rabbit oocytes enriched with sheep decellularized greater omentum

**DOI:** 10.1002/vms3.891

**Published:** 2022-07-27

**Authors:** Khatereh Fazelian‐Dehkordi, Tahereh Talaei‐Khozani, S. Fakhroddin Mesbah A

**Affiliations:** ^1^ Department of Anatomical Sciences Shiraz Medical School, Shiraz University of Medical Sciences Shiraz Iran; ^2^ Histomorphometry and Stereology Research Center Shiraz Medical School, Shiraz University of Medical Sciences Shiraz Iran; ^3^ Tissue Engineering Lab Department of Anatomical Sciences Shiraz Medical School, Shiraz University of Medical Sciences Shiraz Iran

**Keywords:** decellularization, greater omentum, in vitro maturation, oocyte, rabbit

## Abstract

**Background:**

To prevent ovarian hyperstimulation syndrome, in vitro maturation (IVM) allows the oocytes for infertility treatment without hormone therapy. Although many oocytes matured during IVM, some deficiencies in the culture conditions lead to inhibition of the growth and development of the cumulus cells and the oocyte nuclear and cytoplasmic maturation.

**Objectives:**

The challenge of improving the oocyte culture conditions prompted us to use greater omentum (GOM), full of growth factors and proteins, as a rich supplement to the base culture medium.

**Methods:**

Cumulus‐oocyte complexes were recovered from rabbits and divided into 3D and 2D conditions cultured for 12 and 24 h. In 3D cultures, the oocytes embedded in alginate containing FBS decellularized GOM. Corresponding supplements were also added in 2D conditions—maturation of the oocytes evaluated by Aceto‐Orcein, TEM, and RT‐PCR for MAP2K1 and Cdk2.

**Results:**

DNA quantification, Hoechst, and H&E staining confirmed cell depletion from GOM, and SEM showed the preservation of ultra‐architecture after decellularization. Histochemical staining methods showed appropriate extracellular matrix preservation. ELISA assessment showed retention of VEGF content. MTT assessment indicated decellularized GOM was non‐toxic. Both Aceto‐Orcein assessment and ultra‐structure study of the oocytes showed that supplementation of 2D or 3D cultures with decellularized omentum promoted oocyte maturation. Expression of MAP2K1 and Cdk2 also increased in the presence of GOM.

**Conclusions:**

GOM supplementation has a beneficial impact on oocyte maturation, probably due to the presence of growth factors and proteins.

## Graphical abstract

The greater omentum supplementation has a beneficial impact on the oocyte in vitro maturation, probably due to the presence of the growth factors and proteins.

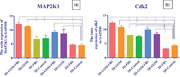

## 1 INTRODUCTION

In recent years, much progress has been made in reproductive technology. The main stage depends on the success rate of the oocyte in vitro maturation. Promoting nuclear and cytoplasmic maturation of the cumulus‐oocyte complex (COCs) is isolated from the immature follicles in the culture medium to get prepared for fertilization is called in vitro maturation (IVM) (Heraud et al., 2017). Before IVM innovation, repeated injections of gonadotropin induce in situ oocytes maturation. Then, the matured oocytes are fertilized in vitro. Compared to traditional in vitro fertilization (IVF), the IVM has many benefits, such as reducing the costs and side effects of using gonadotropin and GnRH analogues (Tao et al., 2008). The best option is the IVM for infertility treatment, selecting the suitable oocyte, abundant production of the oocyte, and the lowest cost in IVF and basic research. However, this method has some disadvantages, including the low growth and development of the surrounding cumulus cells and short maturation of the oocyte nucleus and cytoplasm due to undesirable culture conditions. Challenges to improve IVM have attracted increasing attention to using natural tissues as suitable reserves full of beneficial compounds and nutrition.

First, in vitro rabbit oocyte growth and maturation was performed by Enzmann and Pincus in 1935 (Pincus & Enzmann, 1935). The rabbit oocytes' biochemical and physiological processes have close similarities to humans, so it can be considered an important experimental model to study gamete and embryo engineering (Tao et al., 2008). *Superovulation using gonadotropin* is a process that induces an increase in the number of oocytes in infertile females (Zhang et al., 2017). This process is abundantly used in rabbit models (Zhang et al., 2017). One of the main challenges in assisted reproductive technologies (ART) is avoiding the risk of the ovarian hyperstimulation syndrome (OHSS) due to gonadotropin. Unlike other studies, this study did not use gonadotropin for rabbit COCs recovery.

During the maturation process, both nucleus and cytoplasm maturation are essential. The nucleus is in the metaphase of meiosis II in the mature oocyte. The ooplasm organelles and oocyte maturation were redistributed (Coticchio et al., 2016). Rough endoplasmic reticulum and mitochondria accumulate around the developing mitotic spindle and spread throughout the oocyte in MII (Heraud et al., 2017). Cortical granules originate from the Golgi apparatus at the centre of the immature ooplasm, migrate to the peripheral regions, and close to the oocyte membrane (Lénárt & Ellenberg, 2003). By tracing cortical granules translocation through ooplasm, it is reported that supplementation of vascular endothelial growth factor (VEGF) increased the cytoplasmic maturation (Heraud et al., 2017). Few ultrastructural studies have reported rabbit oocyte maturation in 3D culture (Sarkanen et al., 2012). Therefore, in the present study, the rabbit oocytes' ultrastructural features and morphological characteristics have been evaluated using light and transmission electron microscopy (Shevach et al., 2015).

3D culture mimics in vivo microenvironment and, as a result, the cell morphology, biology and function might be more similar to that in vivo (Hashemi & Soleimani, 2011). 3D scaffolds are considered a substitute for extracellular matrix (ECM) and act as a framework for structural and biochemical support cells (Freyman et al., 2001). The cells attach to the scaffold, proliferate, differentiate, and eventually secrete ECM (Chan & Leong, 2008). Decellularized tissue is used as natural scaffolds in tissue engineering. Greater omentum is one of the most widely used tissues in medicine and tissue engineering (Baumert et al., 2007; Kim et al., 2010; Micheau, 1995; Müller et al., 1992; Ni et al., 2018; Shevach et al., 2014) [M1]. It is composed of adipocyte cells and immune cells called Milky spots (Suh et al., 2004).

It has large blood vessels and various growth factors such as VEGF (Araújo et al., 2014). The beneficial effects of supplementation of the activated GOM Extract were shown previously (Mesbah et al., 2017).

The main aim of this study was to investigate the effects of sheep decellularized GOM in the IVM of the rabbit oocytes. A Xeno culture has been investigated to find a universal IVM system for improving oocyte maturation. Another essential objective of the present study is to recover the oocytes without using hormone therapy to avoid the side effects.

## 2 MATERIAL AND METHODS

### 2.1 GOM decellularization

Fresh GOM of healthy sheep was collected from the nearest slaughterhouse. At first, it was washed with phosphate buffer saline (PBS) and then cut into small pieces (2 ×2  cm). A modified Soffer‐Tsur et al. protocol was used (Soffer‐Tsur et al., 2014). The osmotic shock was done to fresh GOM fragments by incubation in hypotonic buffer containing ten mM Tris and five mM EDTA for 24 h and then dehydration in 70% ethanol and 100% ethanol for 30 min at each stage. They incubated in 100% acetone for 24 h to extract lipids. Tissue pieces were immersed in 100% ethanol for 30 min then incubated overnight in 70% ethanol at 4°C.

Following washing with PBS at pH 7.4, the fragments were incubated in the hypotonic buffer for 2 h. In the main stage, the fragments immersed in 1% SDS dissolved in PBS for 24 h. Another hypotonic shock was performed for 2 h, and the fragments were incubated in 1% SDS for 24 h and then in 2.5 mM sodium deoxycholate for the same time. Traces of detergents were washed by PBS and then washed with 50 mM Tris containing 1 mM MgCl2 at pH 8.0 for one h. Following dehydration was performed again by 70% and 100% ethanol and then by 100% acetone for 30 min. Finally, hexane: acetone (60/40 (v/v)) was used to extract the polar lipids for 24 h. The defatted fragments were rehydrated by ethanol (100 and 70%) for 30 min at 4°C, and then washed three times each in PBS and double‐distilled water.

The decellularized GOM was frozen (−20°C) overnight and lyophilized by a freeze dryer (CHIRST, Alpha 1–2 LD plus, Germany, –50°C). The lyophilized GOM was sterilized by UV light (wavelength: 253.7 nm) for 30 min. Subsequently, the lyophilized GOM (10 mg) were digested with 10 mg of pepsin (1 Anson/gr) (biochemical (BDH), UK) and dissolved in 0.1 ml of hydrochloric acid (HCL (0.1 M), pH 1.6‐2.5) on the shaker for 48–72 h. NaOH (1 N) was used to neutralize the pH. Also, 100 U/ml of penicillin and 100 μg/ml of streptomycin (Gibco) were used to minimize microbial contamination.

### 2.2 Decellularization efficiency

Intact and decellularized GOM were stained with 0.1% Hoechst (33342, Sigma–Aldrich) and haematoxylin and eosin (H&E; Merck, Geneva, Switzerland) to check the nuclear depletion. According to the manufacturer's Guideline, the DNA concentration of the intact and decellularized GOM (*n* = 3) was quantified by dsDNA Assay Kit (QIAGEN, IRAN). In order to evaluate the retention of collagen and elastic fibre retention, Aldehyde fuchsine staining was done on powder decellularized GOM and compared with intact tissue. To estimate the preservation of acidic GAGs and neutral carbohydrates, the samples with Alcian blue and methylene blue were stained (Sigma–Aldrich) at pH = 1 and Periodic acid–Schiff, respectively. Lipid removal was verified by staining the 5‐μm frozen sections of GOMs with Oil Red‐O (Sigma–Aldrich).

### 2.3 Quantitative measurement of VEGF with sandwich‐ELISA

According to the manufacturer's instruction, the concentration of VEGF in the intact and decellularized GOMs was measured by the enzyme‐linked immunosorbent assay kit (ELISA, bioassay technology laboratory).

### 2.4 Scanning electron microscopy

To evaluate the ultra‐architecture of the decellularized GOM, scanning electron microscopy was applied. The sample pieces were fixed with Karnovsky fixative (Sigma‐Aldrich, St. Louis MO, USA) then dehydrated in an increasing graded series of ethanol (50%–100%). They were dried by incubating the samples in a gradually increasing concentration of HMDS. Finally, the pieces were coated with gold by Q150R‐ES sputter coater (Quorum Technologies, UK); they were then observed and imaged by a VEGA3 microscope (TESCAN, Czech Republic).

### 2.5 Oocyte in vitro maturation

#### 2.5.1 Experimental animals

Thirty New Zealand adult white female rabbits, weighing 3 kg ± 0.3 and aged 4–4.5 months, were kept in standard condition (12‐h dark and 12‐h light, 20–24°C and free access to food and water), and 40–60% humidity.

#### 2.5.2 Experimental design oocyte IVM

Oocytes were collected after the sacrifice of donor rabbits. Rabbit COCs were isolated from the immature follicle using two insulin syringe needles with the ‘Slicing’ technique under a stereomicroscope. Then, excellent and good quality COCs were selected and cultured in droplets (50 μl) of Ham's F10 medium (BIO IDEA, Korea) containing FSH (0.1 IU/ml), HCG (5 IU/ml), L‐glutamine, and penicillin (100 IU/ml). The oocytes are divided into 2D groups: 2DGOM‐FBS, 2DGOM, 2DFBS and 2D control and 3D groups: 3DGOM‐FBS, 3DGOM, 3DFBS and 3D control. Each one of them was cultured in a medium containing 10% foetal bovine serum (FBS, Sigma, USA), sodium alginate (0.5 %) or decellularized GOM (0.05 %). In 3D culture conditions, the alginate dissolved in a medium. The oocytes were cultured in alginate droplets without any additive or supplementation of either FBS or decellularized GOM. Then, a droplet of calcium chloride (50 mM) was gently poured on the droplets, turned into a gel, and then incubated at 37.5°C in a humidified atmosphere of 5% CO2 in the air for 12 and 24 h.

#### 2.5.3 MTT assay

The oocytes (*n* = 65) were cultured for 24 h in a medium (DMEM, Sigma, USA) containing 0.5 mg/ml decellularized GOM. Then, MTT solution was added to each well (3 oocytes in each well), and they were incubated for 2 h, then replaced with DMSO and observed by the inverted microscope. The purple oocytes were alive.

#### 2.5.4 Aceto‐Orcein staining

Aceto‐Orcein staining was performed to classify nuclear maturation of the oocytes based on the GV, GVBD, anaphase‐telophase and MII (Kumar et al., 2018). In 3D culture groups, 50 mM sodium citrate was added to degrade the alginate gel to release the oocytes (*n* = 30). Fresh oocytes were also used as the control group (*n* = 30). The oocytes of each group were denuded by 50 μl hyaluronidase (0.5 mg/ml) (Merck, Germany) and pipetting. Then, the oocytes were located on a glass slide and fixed in acetic Acid‐Ethanol (ratio 3‐1) for 24 h. Then, Aceto‐Orcein stain observed oocytes under the Invert microscope, and the nuclear maturation of the oocytes was evaluated.

#### 2.5.5 Evaluation of the COCs ultrastructural

The COCs ultrastructure of various experimental groups was evaluated using TEM. At least three oocytes from each group were sectioned and analysed. The oocytes were fixed for 1 h in glutaraldehyde 2.5%, washed three times in 0.1 mol/L cacodylate buffer, pH 7.4, postfixed in 1% osmium tetroxide for 1 h, washed and gradually dehydrated through ascending the grades of ethanol. Each oocyte was individually embedded in resin by a commercial kit (AGAR, R1031) and sectioned (60–90 nm) on an ultramicrotome (OPTICAL RICHERT, OMU3). The ultrathin sections were mounted on grids and stained with uranyl acetate, followed by lead citrate. The sections were examined using a PHILIPS TEM, CM10TEMat 80 kV.

#### 2.5.6 Real‐time RT‐PCR

Total RNA was isolated from denuded Oocytes (*n* = 20) using the RNX‐Plus kit (cinnagen, RN7713C). The cDNA was synthesized using Add Script cDNA Synthesis Kit (ADD BIO, Korea). The exclusive primers of each marker were designed using Allele ID 7 software (Table 1) and synthesized by Takapoo Zist Tehran Company.

**TABLE 1:** Specifications of primers used in RT‐PCR reaction

Gene	NCBI accession number	Primer forward (5′to 3′) Primer reverse (5′ to 3′)	Annealing temperature (°C)	Amplicon length
Map2k1	NM‐001082629	CTGCTGGATTACATCGTCAA CTGCTTCAAGTCTGCTCTC	60	126
Cdk2	XM‐002711062	GCATTAGAGGCAGGTGAGAG AAAGACAGGAGCAGGGATTC	60	89
GAPDH	NM‐001022531	TGAACCACGAGAAGTATGACAA CCTCCACAATGCCGAAGT	60	114

The primer solution was made at a concentration of 100 pmol/μl. The GAPDH gene is used as a control gene to determine the relative expression of the genes studied. The best temperature of PCR‐Gradient was determined for each gene by Allele ID 7 software (Table 2). The cDNA was synthesized using a kit (Real Q plus Master Mix Green‐low Rox A 3244‐2). The cDNA (2 μl) was added to SYBR green master mix (10 μl), forward (5 μM) and reversed (5 μM) primers.

**TABLE 2:** Thermal protocol for cDNA synthesis

Step	Reaction	Temperature (°C)	Time (min)
1	Primer annealing	25	10
2	Reverse transcription	50	60
3	Heat inactivation	80	5
4	Hold	20	5

#### 2.5.7 Statistical analysis

Statistical analysis was performed using Graph Pad Prism software (Graph Pad, San Diego, CA). All experiments were repeated at least three times independently. One‐way and two‐way analysis of variance (ANOVA) was performed following Tukey's post hoc tests to compare the data. A *p* value less than 0.01 is considered a significant difference.

## 3 RESULTS

### 3.1 Decellularized tissue characterization

**FIGURE 1:**
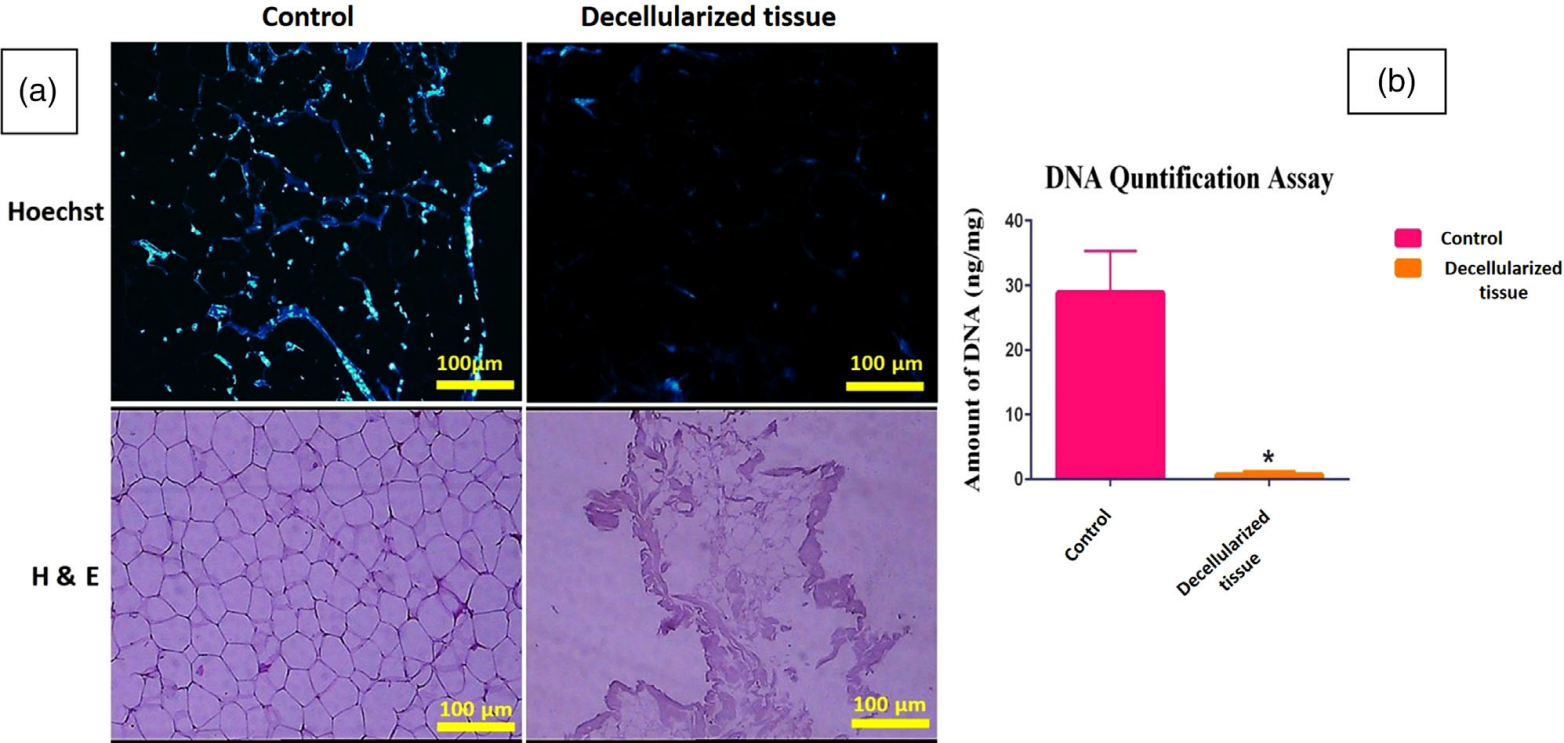
H&E and Hoechst staining: intact tissue (control), decellularized GOM (scale bar = 100 μm) (a). The graph compares DNA quantification after decellularization. Data are expressed as the mean ± standard error of the mean (SEM), *n* = 3 per group (*p* = 0.0251; b)

**FIGURE 2:**
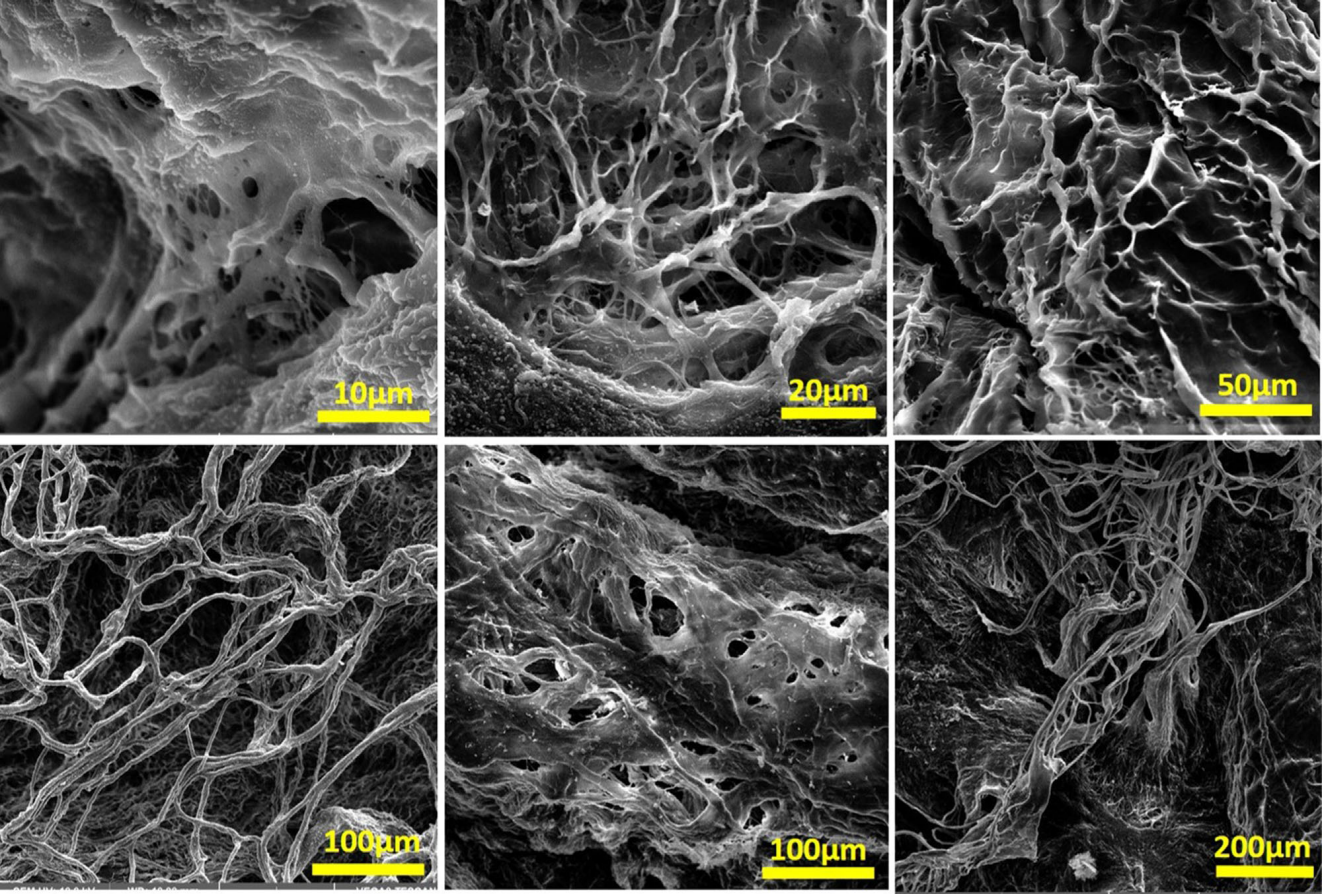
SEM assessment showed ultra‐architecture of the decellularized tissues. They are devoid of cells after decellularization

The H&E staining showed that the honeycomb shape of GOM was destroyed to some extent in the decellularized GOM. This represents that the lipid has been dissolved and extracted in polar and non‐polar solutions. Hoechst staining showed that the decellularized GOM had no nucleus (Figure 1a). DNA quantification test showed a significant decrease in the DNA content after decellularization compared to intact tissue GOM. The amount of DNA was less than 50 ng/mg (standard rate). This amount is enough to prevent immunological reactions after transplantation (Figure 1b). Microphotographs of SEM indicate the network of fibres with pores and ultra‐architecture integrity in decellularized GOM (Figure 2).

**FIGURE 3:**
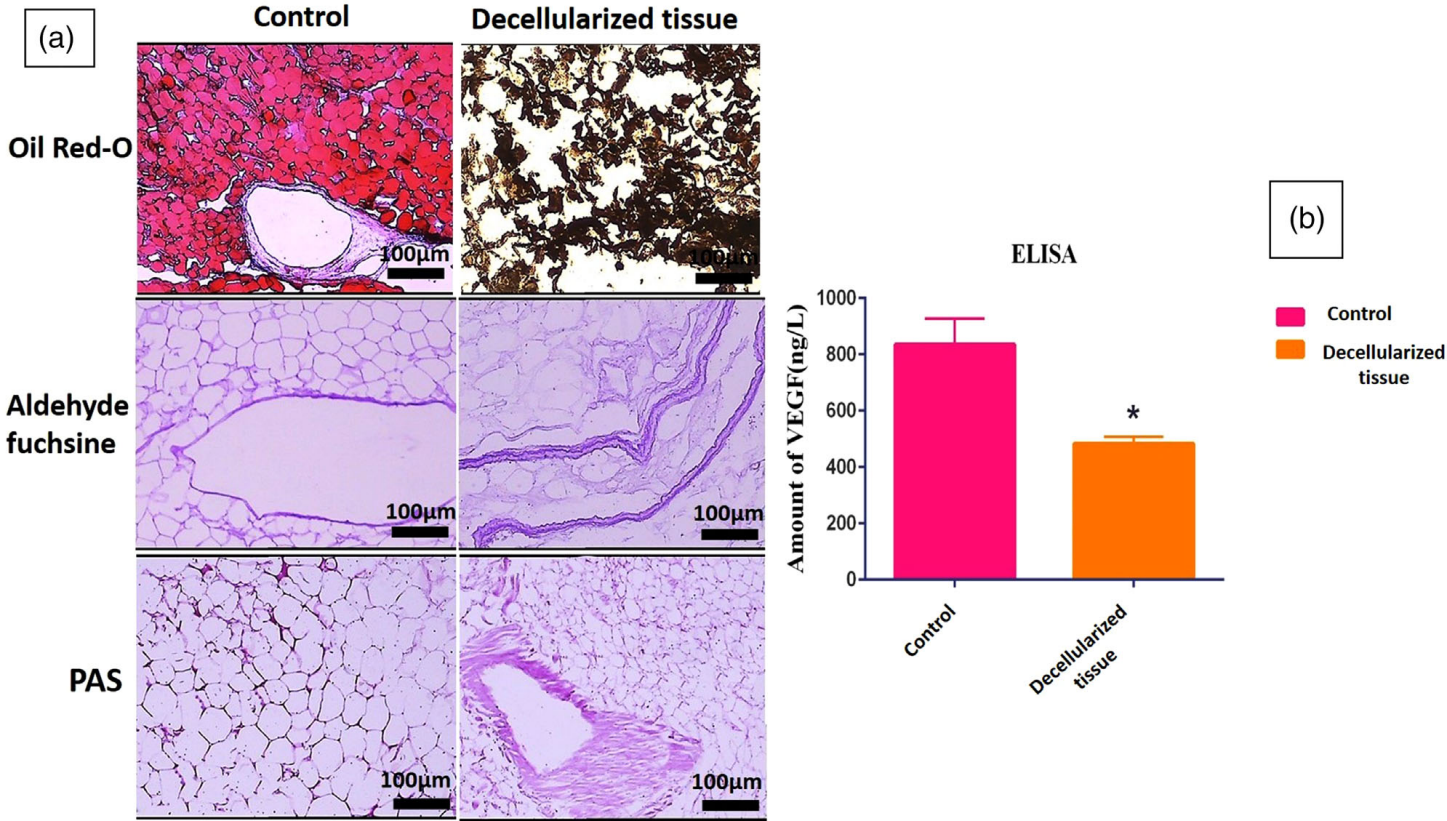
Histochemical assessments of decellularized and undecellularized GOM (scale bar = 100 μm) (a). ELISA assessment showed a significant decrease in the VEGF content after decellularization. Results are presented as mean of VEGF (ng) per Liter dry mass (*n* = 3 per group) (*p* = 0.0346) (b)

**FIGURE 4:**
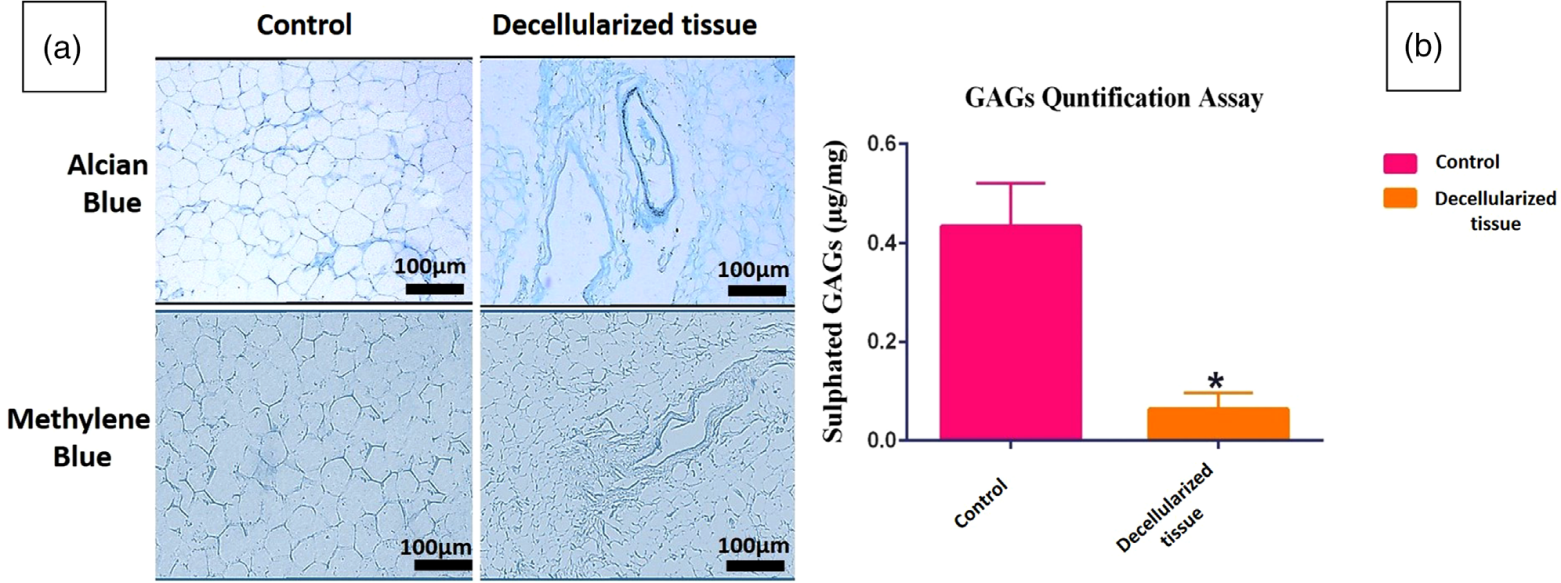
Glycosaminoglycan (GAGs) content assessments. Alcian blue and methylene blue staining of the section of undecellularized tissue and decellularized matrices (scale bar = 100 μm) (a). Quantification of sulphated GAGs showed extensive washing of GAGs by decellularization protocol (*p* = 0.0303) (b)

Aldehyde fuchsine and PAS staining also demonstrated the persistence of collagen and elastic fibres, respectively. Oil Red staining indicated that the decellularization protocol removed the lipid cells acceptably (Figure 3a). Although ELISA assessment showed a significant decrease in the VEGF content after decellularization, it was preserved extensively (VEGF in intact GOM = 900 ng/L and decellularized GOM = 500 ng/L) (Figure 3b). Alican blue and methylene blue staining demonstrated the persistence of GAGs (Figure 4a). Also, the quantification of GAGs showed some extent of its retention in the decellularized GOM compared to the control group (Figure 4b).

### 3.2 Oocyte in vitro maturation

**FIGURE 5:**
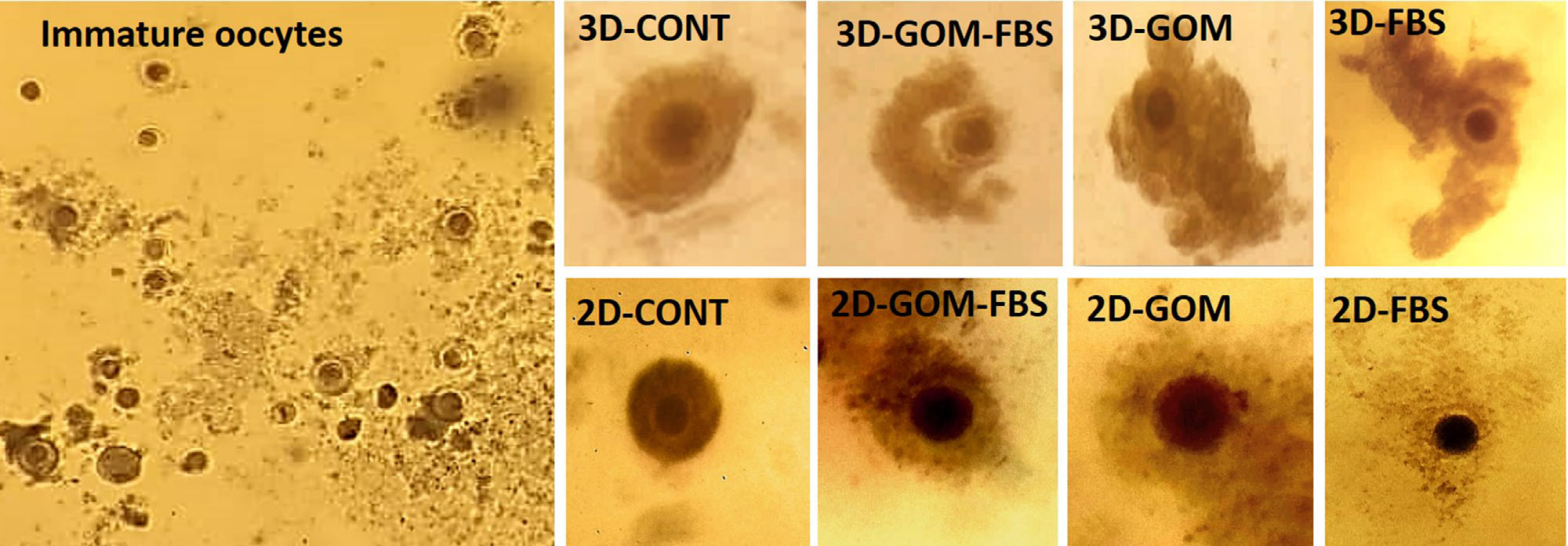
The micrographs of oocytes after collection (immature oocytes) and after IVM in 3D and 2D groups

The micrographs of oocytes after collection and after IVM was showed in Figure 5.

### 3.3 MTT test

**FIGURE 6:**
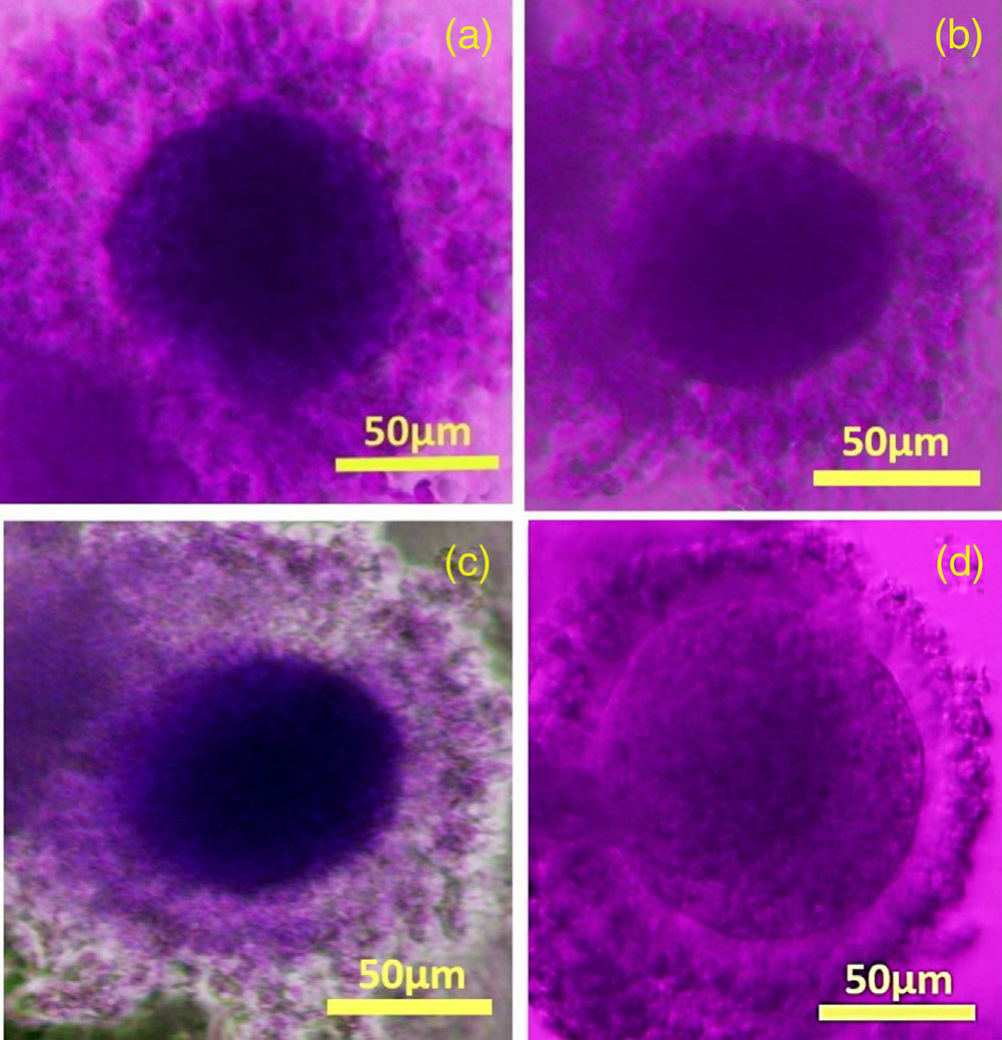
MTT‐stained oocytes after 24 h of IVM; Images a, b and c are live oocytes that are purple colour and 90.77% of the oocytes survived. Image D is a dead oocyte seen in a bright colour (scale bar = 50 μm)

MTT test showed that after 24 h of IVM, out of 65 oocytes, 59 were dark purple, indicating that 90.77% of the oocytes survived (Figure 6).

### 3.4 Aceto‐Orcein staining of the oocytes

**FIGURE 7:**
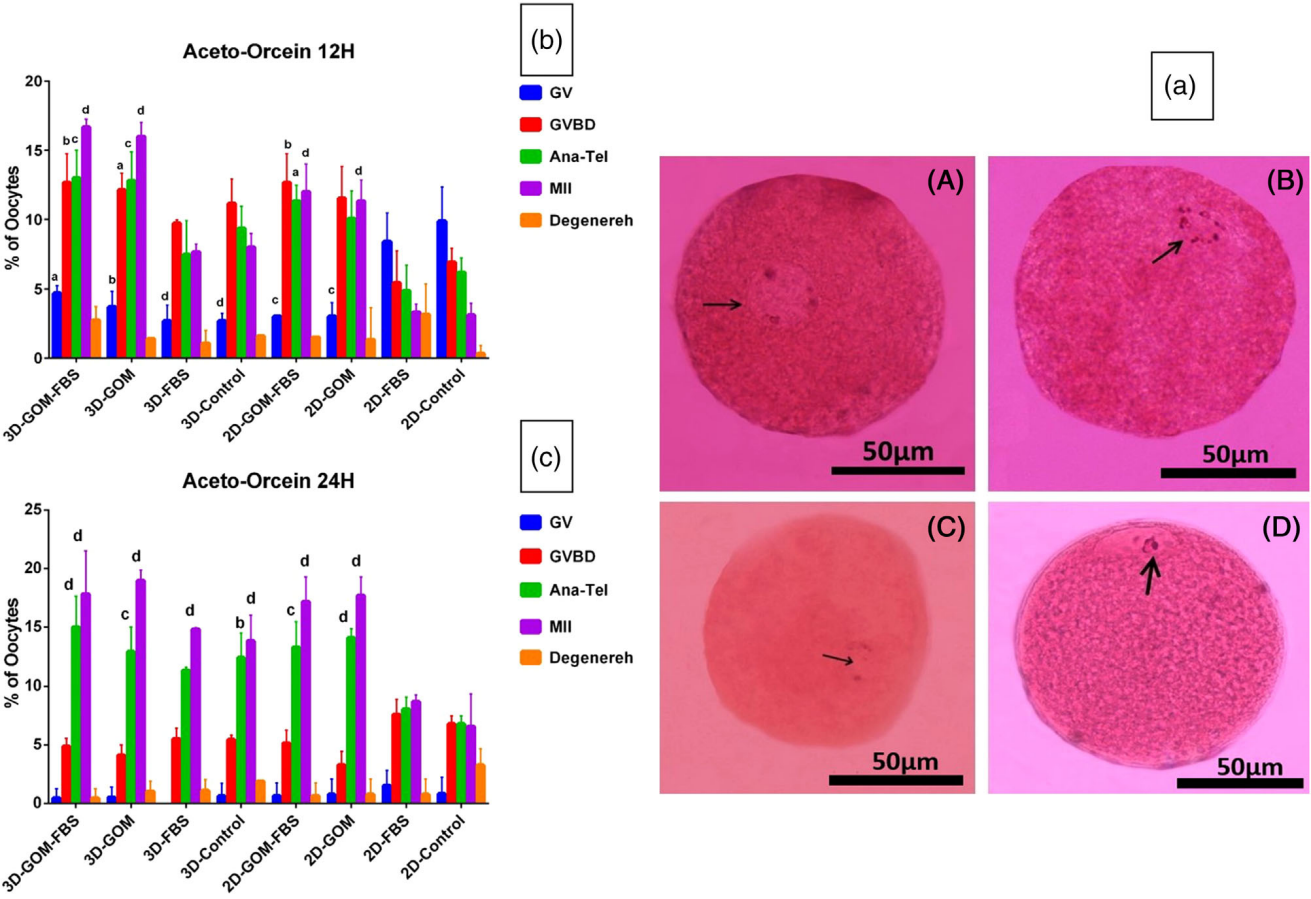
(a) Nuclear maturation with Aceto‐Orcein staining at different times of IVM of rabbit oocytes. (A) GV stage of an oocyte with a nucleus (arrow) containing separate nucleoli and nuclear membranes. (B) Stage of GVBD oocytes without oolema and compressed chromatin (arrows) arranged on the mitotic spindle. (C) Anaphase‐Telophase step in which a pair of homologous chromosomes (arrows) are drawn towards the poles. (D) In the oocyte of stage MII, the arrow points to the first polar body (Scale Bar = 50 μm). (b) Oocyte maturation graph after 12 h with Aceto‐Orcein staining. A significant difference was shown in the control group. (a; *p* ≤ 0.05), (b; *p* ≤ 0.01), (c; *p* ≤ 0.001) and (d; *p* ≤ 0.0001). (c) Oocyte maturation graph after 24 h with Aceto‐Orcein staining assessment. A significant difference was shown in the control group (a; *p* ≤ 0.05), (b; *p* ≤ 0.01), (c; *p* ≤ 0.001) and (d; *p* ≤ 0.0001)

As illustrated in Figure 7a, the oocytes (*n* = 100) showed the GV, GVDB, Ana‐Tel and MII stages of maturation in all groups. After 12 h, a significant increase in the frequency of the oocytes in the GVBD stage was shown in 3D GOM‐FBS (*p* < 0.01), 3D GOM (*p* < 0.05) and 2D GOM‐FBS (*p* < 0.001) compared to 2D control. In addition, a significant increase was observed in 3D GOM‐FBS (*p* < 0.0001), 3D GOM (*p* < 0.001), 3D controls (*p* < 0.01), 2D GOM‐FBS (*p* < 0.0001) and 2D GOM (*p* < 0.01) compared to 2D FBS. A significant increase in the frequency of the oocytes in the Ana‐Tel stage was observed in 3D GOM‐FBS (*p* < 0.0001) and the 3D control group (*p* ≤ 0.001) compared to the 2D control group (Figure 7). The percentage of the oocytes in MII Stage significantly increased in the 3D GOM‐FBS compared to the 3D FBS (*p* ≤ 0.0001), 3D controls (*p* ≤ 0.0001) as well as 2D GOM (*p* ≤ 0.05). An increase in the frequency of the oocytes in MII stage was also observed in 3D GOM, compared to both 3D FBS (*p* ≤ 0, 0001) and 3D control cultures (*p* ≤ 0,001) (Figure 7b). The 96 % of the COCs in 3D culture and 90% of those in 2D culture showed one of the stages of meiotic division (sum of GVBD, Ana‐Tel, and MII) in the groups containing GOM.

After 24 h, the 3D culture condition provided a superior condition for promoting. In the nucleus to Ana‐Tel stages, an increase in percentages of oocytes in this stage was observed in 3D control compared to 2D control groups (*p* < 0.01). The frequency of the oocytes in this stage also significantly increased in 3D GOM‐FBS (*p* ≤ 0.0001), the 3D GOM (*p* ≤ 0.001), 2D GOM‐FBS (*p* ≤ 0.001) and 2D GOM (*p* ≤ 0.0001) compared to 2D control culture. At the same period, the frequency of the oocytes in the MII stage cultured in all the 3D conditions and those cultured in both GOM‐containing 2D conditions was significantly increased compared to the 2D control group (*p* ≤ 0.0001). Also, the percentage of the oocytes at this stage was significantly increased in 3D GOM compared to the 3D control (*p* ≤ 0.05) (Figure 7c). Ninety‐seven per cent of the COCs in 3D culture and 96% of COCs in 2D culture showed one of the stages of meiotic division (sum of GVBD, Ana‐Tel, and MII) in the groups containing GOM.

### 3.5 Quantitative real‐time RT‐PCR assays

**FIGURE 8:**
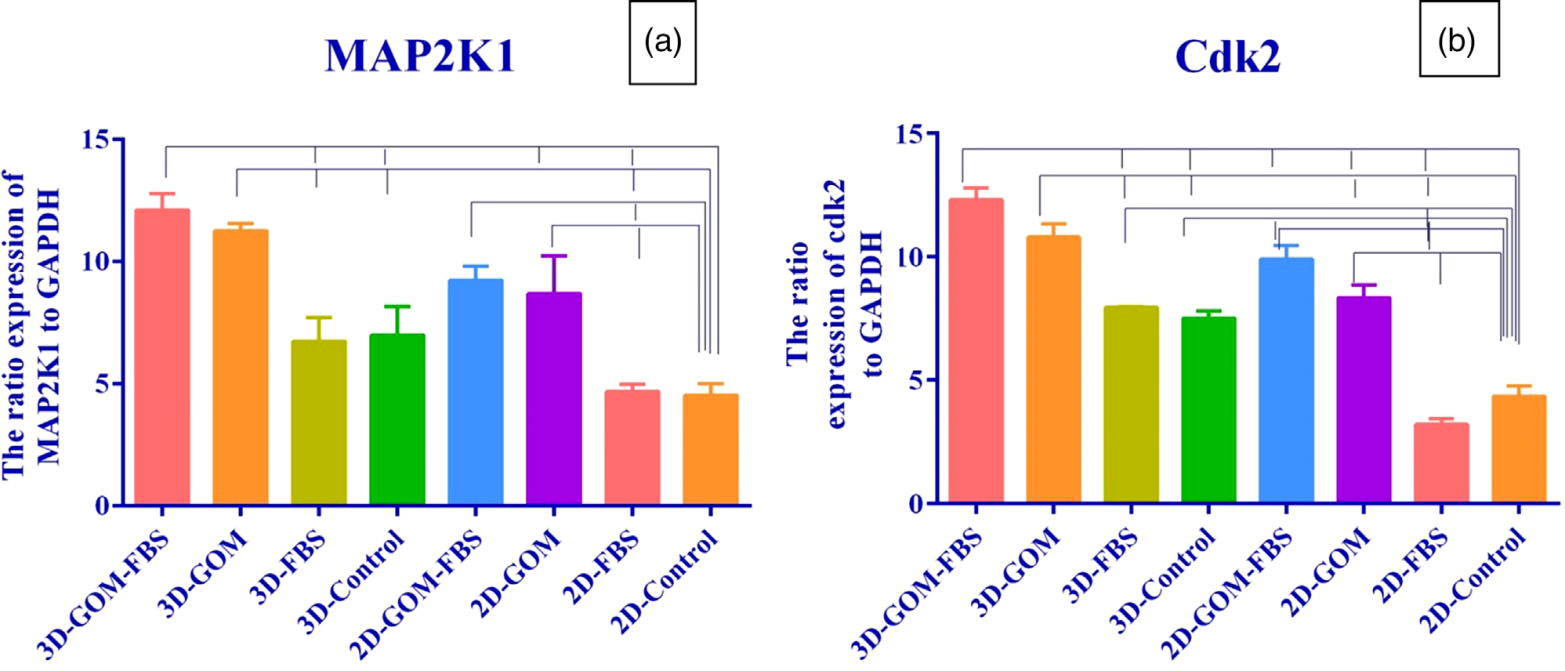
(a) MAP2K1 gene expression graph in rabbit oocytes cultured in 2 and 3‐D culture groups in 24 h (*p* = 0.0001). (b) Cdk2 gene expression graph in rabbit oocytes cultured in 2 and 3D culture groups in 24 h (*p* < 0.0001)

The expression of the MAP2K1 gene, according to the diagram (Figure 8), showed a significant increase in 3D GOM‐FBS and the 3D GOM (*p* ≤ 0.001), and 2D GOM‐FBS (*p* ≤ 0.01) and 2D GOM (*p* ≤ 0.05) compared to 2D control cultures. GOM also showed a synergistic impact with FBS on the expression of MAP2K1 as the expression of this gene was significantly more in 3D GOM‐FBS than 3D‐FBS (*p* ≤ 0.01). The expression level of MAP2K1 was also higher in 3D GOM‐FBS groups compared to the 3D control (*p* ≤ 0.01), 2D GOM (*p* ≤ 0.05), and 2D FBS (*p* ≤ 0.001). The oocytes matured in the 3D GOM condition showed an elevation in gene expression compared to the 3D control (*p* ≤ 0.05) and the 2D FBS (*p* ≤ 0.001) (Figure 8a).

The expression of the Cdk2 was significantly higher than 3D GOM‐FBS and the 3D GOM (*p* ≤ 0.0001), 3D FBS, 3D controls, 2D GOM (*p* ≤ 0.01), and 2D GOM‐FBS (*p* ≤ 0.001) compared to the 2D control condition. The most expression level of Cdk2 was observed in both 3D and 2D conditions containing GOM. In addition, all 3D groups containing GOM showed significantly more gene expression than 3D groups without GOM (Figure 8b).

### 3.6 The COCs ultrastructural observations

**FIGURE 9:**
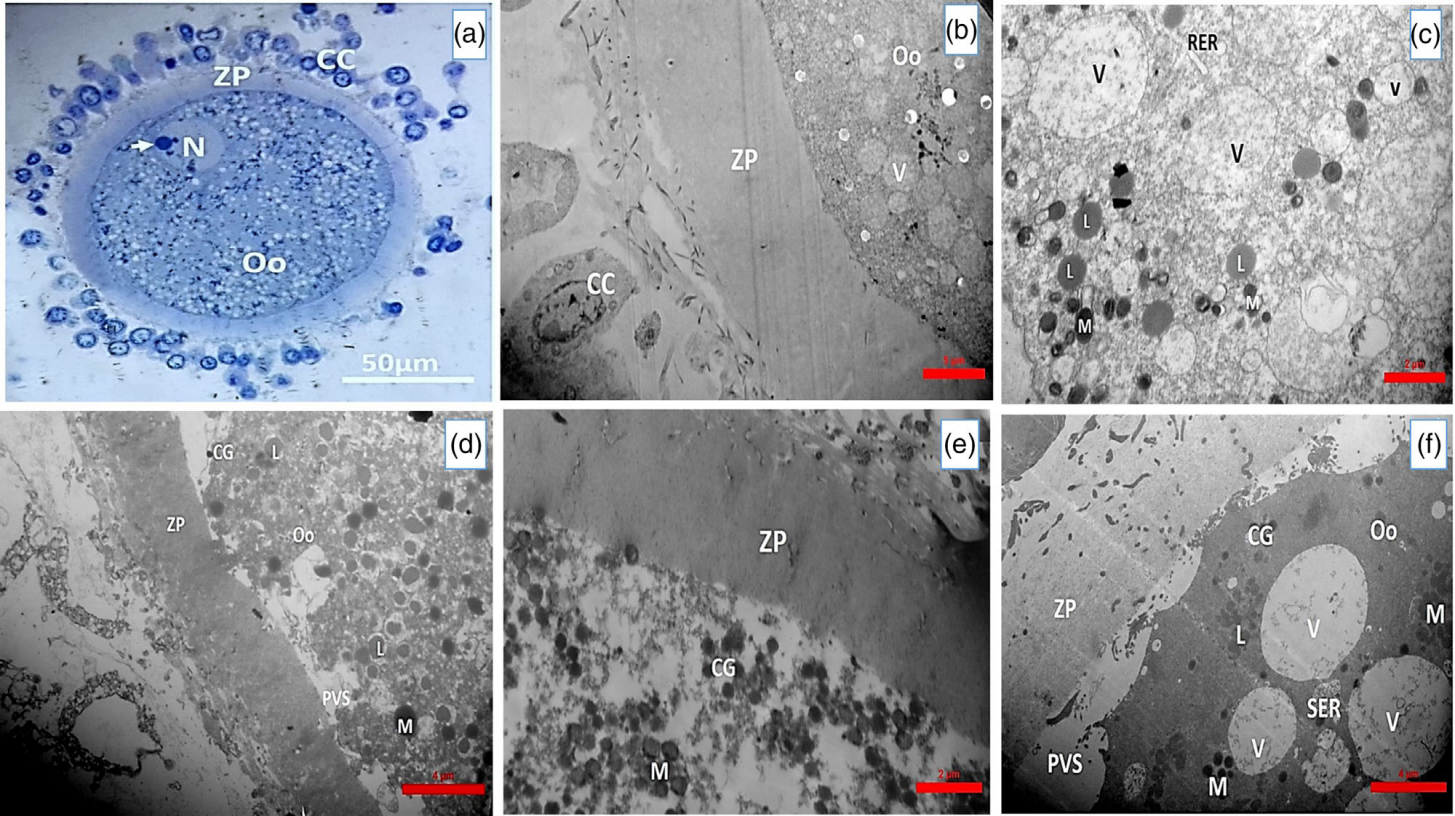
(a) A light micrograph of an immature rabbit oocyte staining with toluidine blue (scale bar = 50 μm). A transmission electron micrograph of (b) an immature rabbit oocyte (scale bar = 5 μm). (c) 3D culture groups 12 h of oocyte (scale bar = 2 μm). (d) 2D culture groups 12 h of oocyte (scale bar = 4 μm), (e) 3D culture groups 24 h of oocyte (scale bar = 5 μm), (f) 2D culture groups 24 h of oocyte (scale bar = 4 μm) that show cumulus cell (CC), zona pellucida (ZP), perivitelline space (PVS), and ooplasm that contain vacuoles (V), mitochondria (M), lipid droplet (L), cortical granule (CG), smooth endoplasmic reticulum (SER) and nucleus (N)

The results of the COCs ultrastructural are summarized in Table 1. The control group results showed that immature Rabbit oocytes were spherical and surrounded by multilayers of compact cumulus cells containing regular features patterns of cytoplasmic organelles; however, a few pyknotic cells were also observed. ZP was an intact and uniform transparent layer surrounding the oocyte. In the majority of the oocytes, the perivitelline space (PVS) was narrow. The ooplasm was homogeneous and contained lipid droplets, vacuoles, vesicular RER and mitochondria distributed throughout the ooplasm. The nucleus in the GV stage was peripherally located in all oocytes (Figure 9a).

Changes in the distribution and number of organelles in the GOM‐containing groups compared to the control groups in the 12‐ and 24‐h 2D and 3D culture groups are summarized in Table 3. Also, TEM images of 2D and 3D culture groups are shown in the image (Figure 9b–e).

**TABLE 3:** COCs Ultra structural observations. The changes in the distribution of organelles and their number in the 2D and 3D culture groups at 12 and 24 h, in the groups containing GOM compared to the control groups

Organelles	Immature oocyte (Control groups)	Mature oocyte 2D groups	Mature oocyte 3D groups
Granulosa cell	Multilayer compact	Multilayer expanded	Multilayer expanded
ZP	All	All	All
PVS	None	Few	Dilated
Nucleus	Eccentric	Undetectable	Undetectable
Mitochondria	Many round	Many all over	Many all over
Lipid droplet	Few	Many	Many
Vesicle	Few	Many polymorphism	Many polymorphism
ER	Many	Few	Few
Golgi	Many	Few	Few
Cortical granules	Few	Many	Many

## 4 DISCUSSION

In the present study, we used 3D Xeno culture of sheep GOM to improve oocytes IVM in rabbits for the first time.

The current study data showed that decellularized GOM contained collagen, GAGs, and proteins such as VEGF. We showed that the decellularized GOM provided a suitable microenvironment for the maturation of the nucleus and ooplasm of the oocytes. IVM is the first and most crucial step to successful IVF. The culture medium, proteins, and hormones (FSH, LH,) supplements for IVM play an essential role in the maturation of the oocyte (Bavister et al., 1992). However, adverse cultivation conditions lead to insufficient growth and development of the oocytes in vitro. This study showed that the nuclear and ooplasm maturation was higher in the oocytes that received GOM than the others. Some of the animals should be super‐ovulated to do IVM. The first attempt to induce ovulation in rabbits using synthetic GnRH analogues was made 20 years ago. Subsequent studies have shown successful ovulation by intravenous administration of HCG and intramuscular administration of GnRH (Molina et al., 1987). In most studies on IVM of rabbit oocytes, superovulation has been performed (Viudes‐de‐Castro et al., 2017) by injection of hormonal drugs such as HCG (Izumi et al., 2013), FSH (Zhang et al., 2017), and PMSG (Izumi et al., 2013) to recover the COCs. Unfortunately, superovulation in humans has many serious side effects, such as ovarian hyperstimulation syndrome. We did not use any gonadotropin for rabbit oocyte recovery in the present study.

The culture medium for IVM is considered a key element in the success rate of this technique. In the present study, Ham's F10 medium was used for the first time in rabbit oocyte culture; other studies have used TCM‐199 (Arias‐Álvarez et al., 2018; Zhang et al., 2017). Ham's F10 medium is not as rich and nutritious as the TCM‐199 culture medium. Since the GOM is rich in nutrients, proteins, and growth factors, it enriches the culture medium. According to the results obtained in all the 2D and 3D culture groups containing the GOM, more oocytes matured than the 2D control group.

VEGF and its receptors are present in many cell types, including granulosa cells and follicles. Maximum levels of VEGF in the follicular fluid have been reported in bovine pre‐ovulatory oocytes, which express both VEGF receptors and granulosa cells (Ackbar et al., 2012). In addition, VEGF‐A may indirectly lead to increased storage of growth factors, gonadotropin, steroids, and oxygen by increasing cell permeability (Danforth et al., 2003). One of the mechanisms of action of VEGF is that this factor leads to an increase in intracellular glutathione during the maturation of pig oocytes (Barnes et al., 2011). Glutathione is necessary for the proliferation of the granulosa cells (Azuma et al., 2007) and the maintenance of the mitotic spindle (Zuelke et al., 2003). VEGF has also been shown to increase the maturity of the oocytes isolated from small follicles (Bui et al., 2017), rate of blastocyst formation (Anchordoquy et al., 2015), completion of the second meiotic division (Yan et al., 2012), and rate of fertilization (Biswas & Hyun, 2011). Mechanisms to increase the maturation of the oocytes in GOM‐treated groups may be due to VEGF content. VEGF interacts with receptors in the granulosa cells and acts as a mitogenic factor in the growth of the preantral follicles in humans (Abir et al., 2010). Bovine granulosa cells express VEGF‐A mRNA and receptors (Yang & Fortune, 2007). In the present study, in the decellularization of GOM, VEGF has been mainly preserved. The use of GOM in IVM causes an increase in the growth and development of maturation in the cultured oocytes.

The control group results showed that most immature rabbit oocytes, like other mammals, were spherical. A few oocytes were in the oval, which could be due to mechanical pressure during the preparation of the resin block. The phenotype of the most cumulus cells in this study was similar to other studies performed on the rabbit oocytes, i.e., spherical and round (Zamboni & Mastroianni, 1966). Compression of the cumulus cells and their close contact with the oocytes was a common feature of immature oocytes in various mammalian species (De Loos et al., 1989). The presence of dense cumulus cells, GV nuclei and uniform ooplasm in immature oocytes indicates the ability to resume meiotic division and readiness for puberty (Dell'Aquila et al., 1996). PVS was narrow in some oocytes and dilated in culture groups due to rupture of junctions between the oocyte and the cumulus cells, indicating that the oocytes had matured. Cortical granules are seen throughout the ooplasm of immature oocytes, while in mature oocytes, they were placed in the cortical area of the ooplasm.

Significant variation in the size and number of lipid droplets observed in this study is a similar feature in the oocytes of other mammals (Assey et al., 1994). In addition, the distribution of lipid droplets in the middle part of the ooplasm is one of the characteristics of immature oocytes; in mature oocytes, they are seen at the periphery. These droplets are the source of cell energy. In immature oocytes, a close association was observed between the mitochondria, membranous vesicles, RER, and lipid droplets in the ooplasm, which indicates the cell's need for metabolism and high‐energy demand. In the 3D culture groups containing the GOM in 12 and 24 h, the number of lipid droplets decreased compared to the two‐dimensional groups. Mitochondrial activity and distribution are associated with oocyte development and growth. This organelle is the primary source of intracellular energy and produces energy through the oxidative phosphorylation of ATP (Arias‐Álvarez et al., 2018). Mitochondria were observed in 2D and 3D culture groups containing GOM at 12 and 24 h of culture, mainly in the round and complex form in the cortical area of the ooplasm. Dispersion of the mitochondria throughout the ooplasm, reducing its association with vacuoles and nuclear membranes, and reducing long and dumbbell‐shaped mitochondria indicate metabolic changes in the oocyte and its readiness for full maturity and fertilization (Anchordoquy et al., 2015).

Based on the results of Aceto‐Orcein staining, the first polar body was seen more in the 3D groups containing GOM than other groups at 12 h of culture, which increased in 24 h. In addition, the MII stage in the oocytes of 2D culture containing GOM was shown more than the 2D control group.

In the present study, more nuclear maturation was observed in 12 and 24 h 3D culture groups containing GOM compared to the 3D control group. Various studies on alginate have shown that the culture of preantral follicles of rats with alginate hydrogel can produce fertilized oocytes (Jin et al., 2010). Mice follicles encapsulated with 0.5% sodium alginate showed the highest survival, development, and maturity (Abdi et al., 2013). In the present study, the GOM‐alginate combination provided a better condition for the maturation and development of rabbit oocytes.

This study revealed that GOM supplementation in both 2D and 3D conditions had a superior impact on MAP2K1 expression as its replacement with FBS led to a higher level of gene expression than that in the FBS‐ treated groups.

## 5 CONCLUSION

This study showed that GOM was successfully decellularized, with good collagen and elastic fibres preservation in histochemical staining, GAGs, VEGF and proteins in the ECM. The in vitro maturation of oocytes showed that the 3D GOM culture condition provided a batter microenvironment for the maturation of the oocyte. It may be due to the richness of GOM in the nutrients, growth factors, and proteins, and this solution can be suggested as beneficial supplementation to accelerate IVM (12 h). In addition, we successfully obtained the rabbit COCs without hormonal drugs in the present study. This is important in reducing the widespread side effects of using hormonal drugs in ART and the threatening risks to infertile women.

## CONFLICT OF INTEREST

The authors declare no conflict of interest.

## ETHICS STATEMENT

This project was approved by the ethics committee of Shiraz University of Medical Sciences (IR.SUMS.REC. 1396.S1013).

## AUTHOR CONTRIBUTIONS

KFD: involved in data collection, data analyses, and drafting of the manuscript; TTK: involved in experimental design, data analyses, revising the manuscript, and supervision; SFMA, involved in idea development, experimental design, data analyses, fund acquisition, and supervision.

### PEER REVIEW

The peer review history for this article is available at https://publons.com/publon/10.1002/vms3.891.

## Data Availability

The data of manuscript are available.
